# Prevalence and incidence of clinical outcomes in patients presenting to secondary mental health care with mood instability and sleep disturbance

**DOI:** 10.1192/j.eurpsy.2020.39

**Published:** 2020-04-27

**Authors:** Keltie McDonald, Tanya Smith, Matthew Broadbent, Rashmi Patel, John R. Geddes, Kate E. A. Saunders

**Affiliations:** 1 Department of Psychiatry, University of Oxford, Warneford Hospital, Oxford OX3 7JX, United Kingdom; 2 Division of Psychiatry, UCL, London, W1T 7NF, UK; 3 Oxford Health NHS Foundation Trust, Oxford OX3 7JX, United Kingdom; 4 South London and Maudsley NHS Foundation Trust, Biomedical Research Center Nucleus, London SE5 8AF, United Kingdom; 5 Institute of Psychiatry, Psychology and Neuroscience, King’s College London, London WC2R 2LS, United Kingdom

**Keywords:** Electronic health records, epidemiology, mood, psychiatry, sleep

## Abstract

**Background.:**

Mood instability and sleep disturbance are common symptoms in people with mental illness. Both features are clinically important and associated with poorer illness trajectories. We compared clinical outcomes in people presenting to secondary mental health care with mood instability and/or sleep disturbance with outcomes in people without either mood instability or sleep disturbance.

**Methods.:**

Data were from electronic health records of 31,391 patients ages 16–65 years presenting to secondary mental health services between 2008 and 2016. Mood instability and sleep disturbance were identified using natural language processing. Prevalence of mood instability and sleep disturbance were estimated at baseline. Incidence rate ratios were estimates for clinical outcomes including psychiatric diagnoses, prescribed medication, and hospitalization within 2-years of presentation in persons with mood instability and/or sleep disturbance compared to individuals without either symptom.

**Results.:**

Mood instability was present in 9.58%, and sleep disturbance in 26.26% of patients within 1-month of presenting to secondary mental health services. Compared with individuals without either symptom, those with mood instability and sleep disturbance showed significantly increased incidence of prescription of any psychotropic medication (incidence rate ratios [IRR] = 7.04, 95% confidence intervals [CI] 6.53–7.59), and hospitalization (IRR = 5.32, 95% CI 5.32, 4.67–6.07) within 2-years of presentation. Incidence rates of most clinical outcomes were considerably increased among persons with both mood instability and sleep disturbance, relative to persons with only one symptom.

**Conclusions.:**

Mood instability and sleep disturbance are present in a wide range of mental disorders, beyond those in which they are conventionally considered to be symptoms. They are associated with poor outcomes, particularly when they occur together. The poor prognosis associated with mood instability and sleep disorder may be, in part, because they are often treated as secondary symptoms. Mood instability and sleep disturbance need better recognition as clinical targets for treatment in their own right.

## Introduction

Mood instability (MI) and sleep disturbance (SD) are clinically important features of mental illness. Although part of the diagnostic criteria for only some conditions, they are present in a wide range of mental disorders and have been regarded individually as potential transdiagnostic processes in the origin and maintenance of mental illness [1–3]. MI and SD may be involved in a set of pathways that interact to produce poor mental health outcomes that transcend diagnostic boundaries [4,5]. However, clinical evidence of the relationship between MI and SD is limited, and research has been mostly confined to small samples of specific mental disorders with limited generalizability.

We have previously demonstrated that MI and SD are closely linked and common in the general population, irrespective of mental illness [6]. MI and SD are associated with extensive use of resources for mental health care, and their co-occurrence may be related to poorer health and social outcomes than either symptom alone [7].

To further investigate the relationship between MI and SD as transdiagnostic features within the clinical population, this study examined data from a large sample of electronic health records (EHRs) from secondary mental health care. In the United Kingdom, access to secondary mental health care providers of specialized services such as hospitals, some psychological services, community mental health teams, and early intervention teams. Usually requires a referral from primary health care providers (such as general practitioners [GPs]). EHRs contain a wealth of clinical information from secondary services, often recorded as text. Recently, the development of novel natural language processing (NLP) approaches has enabled efficient identification of clinical information from text within EHRs, thereby permitting the secondary analysis of large quantities of data not typically feasible by manual review. We used NLP to study MI and SD in a large sample of individuals presenting to mental health services. Specifically, we sought to describe the demographic and clinical features of persons presenting with MI and/or SD and to assess the incidence rates of clinical outcomes in individuals presenting with MI and/or SD relative to persons with neither symptom.

## Methods

### Data source

Data were obtained from EHRs from the Oxford Health NHS Foundation Trust (OHFT) using the Clinical Research Interactive Search (CRIS; https://crisnetwork.co.uk). OHFT is the National Health Service (NHS) provider of mental health care to a catchment area of approximately 1.6 million residents in Oxfordshire, Buckinghamshire, Swindon, Wiltshire, Bath, and North East Somerset. CRIS is an anonymized database of EHRs developed at South London and Maudsley (SLaM) NHS Foundation Trust and King’s College London with the National Institute for Health Research (NIHR), and consists of structured (e.g., numbers and dates) and unstructured text (e.g., clinical notes and correspondence) data fields.

### Sample

We included EHRs of all individuals aged 16–65 years who presented to the OHFT between December 31, 2007 and January 1, 2016. Study entry was the date of the patients’ first clinical progress note. Patients were followed-up until the date of their most recent clinical progress note or 31 January 2018. Based on the assumption that patients hospitalized long-term are not representative of the broader clinical psychiatric population, we excluded patients with a hospital duration longer than two standard deviations above the mean number of days in hospital for the total patient sample (mean = 19.8, SD = 120.1 days).

### Data extraction

Where available, data were extracted from routinely completed structured fields. Data extraction from unstructured fields was supported by a set of NLP applications described previously [8,9]. These applications permit efficient, automatic processing of free text clinical notes and correspondence using General Architecture for Text Engineering (GATE) [10] and TextHunter [11] software.

### Measures

#### Mood instability

Three NLP applications were used extract documentation of MI, affective instability, and emotional instability, and outputs were combined into a single parameter (which we termed MI). The terms identified by the applications included frequently used combinations of mood, affect, and instability with modifier terms such as dysfunction, instability, and lability [8].

#### Sleep disturbance

The presence of sleep problems and insomnia were identified using two separate NLP applications. The sleep problems application searched for instances of selected modifier terms (poor, interrupt, disturb*, inadequate, no, problem*) within 0–5 words either side of the keyword (sleep). The insomnia application searched for instances of insomn* within the text. Since the NLP applications for insomnia and sleep problems were produced separately, we initially evaluated their outputs separately. However, given the overall aim of assessing SD more generally, we combined their outputs into a single SD variable for the main analysis.

#### Prescribed medication

The medication application was designed to extract the names and dosages of medications prescribed to the patient. The application ignores medications that might be prescribed in the future (e.g., a medication to be considered if the patient’s condition worsens). We included antidepressants, antipsychotics, mood-stabilizing antiepileptics, lithium, sedative-hypnotics, sympathomimetics, amphetamines, and sedating antihistamines, defined according to the British National Formulary (https://bnf.nice.org.uk/; Supplementary Table S1).

#### Diagnosis

Diagnoses, classified according to the International Classification of Disease 10th revision (ICD-10) [12] were extracted from structured fields, including primary diagnoses of organic mental disorders (F00–F09), substance use disorders (F10–F19), schizophrenia and related (F20–F29), mood disorders (F30–F39), anxiety disorders (F40–F49), behavioral syndromes (F50, F52–F59), personality disorders (F60–F69), mental retardation (F70–F79), disorders of psychological development (F80–F89), behavioral and emotional disorders with onset usually in childhood and adolescence (F90–F98), and unspecified mental disorders (F99). Given the specific interest in SD, sleep disorders (F51, G47) were initially examined separately, but sample sizes were too small for estimation.

#### Events

The number of contacts (events) for each patient was identified from dates of clinical notes, diary and clinic appointments, and participation in group therapies.

#### Hospitalization

Frequency and duration of hospitalization were extracted based on hospital admission and discharge dates recorded in structured fields.

#### Health of the Nation Outcome Scale

The Health of the Nation Outcome Scale (HoNOS) is a 12-item scale that measures the health and social functioning of people of working age with severe mental illness [13]. HoNOS is a routine outcome measure for the NHS used to assist with offering patients the care and interventions to meet their individual needs [14]. The total score was adjusted for missing responses to individual items (12*total score/number of responses). Assessments missing more than three responses were excluded.

### Statistical analysis

We selected a random subset of 100 documents from the final sample to validate each NLP application against classifications by a human annotator (K.M.). Each feature (MI, sleep problems, insomnia, and medication) was classified as present or absent at the document-level. Manual classifications were compared with the NLP classifications, and sensitivity, and positive predictive value were calculated.

For the remaining analyses, study groups (MI-only, SD-only, MI and SD, no MI or SD) were defined based on their documentation within 1 month of first presentation to OHFT (baseline). This enabled comparability with a methodologically similar study [8] and was compatible with timings of outcomes of interest. Prevalence was calculated as proportions with 95% confidence intervals (CI) at baseline and 2-years follow-up. Demographic and clinical features within each subgroup were described using estimates of frequencies, proportions, means and standard deviations. Incidence rate ratios (IRRs) of outcomes in each study group (MI-only, SD-only, and MI and SD) versus the common referent (no MI or SD) were assessed within 2-years of presentation. The family-wise error rate was controlled using the Bonferroni–Holm procedure [15]. All analyses were performed in R [16].

## Results

A total of 31,912 patients aged 16–65 years presented to OHFT between December 31, 2007 and January 1, 2016. We excluded 521 patients for having a hospital duration greater than 260 days (see Section “Sample”), yielding a final sample of 31,391 patients.

### Validation of the NLP outputs

Estimates of sensitivity ranged between 70.3% for MI and 85.9% for the medication application. Positive predictive values ranged between 82.1% for sleep problems and 93.8% for the MI application (Table 1).

**Table 1.** Estimates of sensitivity and positive predictive value for each NLP classifier against manual annotation.

	Mood Instability		Sleep Problems		Insomnia		Medication	
	%	95% CI	%	95% CI	%	95% CI	%	95% CI
Se	70.31	57.58–81.09	80.00	64.35–90.95	84.00	70.89–92.83	85.94	74.98–93.36
PPV	93.75	82.80–98.69	82.05	66.47–92.46	89.36	76.90–96.45	91.67	81.61–97.24

Abbreviations: PPV, positive predictive value; Se, sensitivity.

### Demographic and clinical features of the sample at baseline and 2-years follow-up

Of the total sample, approximately 4.2% (*n* = 1,322) had MI-only, 20.9% (*n* = 6,559) had SD-only, 5.4% (*n* = 1,684) had MI and SD, and 69.7% (*n* = 21,826) had neither symptom documented at baseline.

Patients presenting with SD-only were more often male, whereas the remaining groups were more often female (Table 2). Age distributions were similar across all groups. Patients presenting with MI and SD were most often diagnosed with any psychiatric (29.9%), mood (16.3%), personality (4.6%), and schizophrenia and related (3.4%) disorders. MI-only showed the highest proportion of behavioral syndromes (1.9%), whereas SD-only had the highest proportion of anxiety disorders (5.5%). Few individuals without either symptom had any psychiatric diagnosis (11.7%).

**Table 2.** Descriptive features of the sample with mood instability-only, sleep disturbance-only, mood instability and sleep disturbance, and no mood instability or sleep disturbance within 1 month of presenting to OHFT (*n* = 31,391).

	Mood instability-only		Sleep disturbance-only		Mood instability and sleep disturbance		No mood instability or sleep disturbance	
	(*n* = 1,322)		(*n* = 6,559)		(*n* = 1,684)		(*n* = 21,826)	
	*n*	%	*n*	%	*n*	%	*n*	%
Age								
16–25	164	12.41	567	8.64	176	10.45	2,122	9.72
26–35	426	32.22	1,966	29.97	578	34.32	6,629	30.37
36–45	360	27.23	1,907	29.07	473	28.09	5,936	27.20
46–55	315	23.83	1,703	25.96	360	21.38	4,707	21.57
56–65	57	4.31	416	6.34	97	5.76	2,432	11.14
Gender, female	761	57.56	3,408	51.96	939	55.76	12,096	55.42
Diagnosis								
Any	231	17.47	1,460	22.26	503	29.87	2,560	11.73
Organic	<5	–	11	0.17	<5	–	71	0.33
Substance use	24	1.82	143	2.18	53	3.15	166	0.76
Schizophrenia and related	29	2.19	208	3.17	58	3.44	470	2.15
Mood	90	6.81	719	10.96	274	16.27	919	4.21
Anxiety	48	3.63	361	5.5	88	5.23	484	2.22
Behavioral syndrome	25	1.89	83	1.27	19	1.13	346	1.59
Personality	37	2.80	120	1.83	78	4.63	254	1.16
Mental retardation	<5	–	<5	–	<5	–	<5	–
Developmental	<5	–	5	0.08	<5	–	32	0.15
Behavioral/emotional	<5	–	9	0.14	<5	–	43	0.20
Unspecified	<5	–	22	0.34	12	0.71	61	0.28
Medication								
1	283	21.41	1,241	18.92	268	15.91	1,990	9.12
2	208	15.73	1,063	16.21	243	14.43	1,171	5.37
3+	232	17.55	1,944	29.64	836	49.64	1,038	4.76
Any	723	54.77	4,248	64.77	1,347	79.99	4,199	19.24
Antidepressant	475	35.93	3,158	48.15	901	53.50	2,973	13.62
Antipsychotic	255	19.29	1,529	23.31	642	38.12	1,395	6.39
Mood-stabilizing antiepileptic	62	4.69	181	2.76	114	6.77	225	1.03
Sedative hypnotic	183	13.84	1,597	24.35	673	39.96	912	4.18
Lithium	26	1.97	86	1.31	57	3.38	115	0.53
Sedating antihistamine	6	0.45	57	0.87	32	1.90	20	0.09
Sympathomimetic	<5	–	21	0.32	7	0.42	42	0.19
Amphetamine	<5	–	<5	–	<5	–	6	0.03
Events								
12–23	117	8.85	493	7.52	59	3.50	4,694	21.51
24–35	159	12.03	658	10.03	99	5.88	4,466	20.46
36+	1,002	75.79	5,200	79.28	1,449	86.04	11,777	53.87
Hospitalization								
Days in hospital, mean (SD)	89	17.40 (11.04)	580	14.73 (10.72)	429	16.92 (10.15)	374	16.43 (11.67)
1	88	6.66	560	8.54	408	24.23	363	1.66
2	<5	–	20	0.30	20	1.19	11	0.05
3+	<5	–	<5	–	<5	–	<5	–
HoNOS, mean (SD)	69	10.45 (5.16)	377	11.94 (6.67)	114	12.12 (6.10)	427	11.02 (5.87)

Abbreviation: HoNOS, Health of the Nation Outcome Scale adjusted total score.

– represents estimates unavailable due to small numbers.

Polypharmacy was most frequent in patients with both MI and SD, whereas patients without MI or SD were taking the fewest medications. Patients with MI and SD (24.2%) were most often hospitalized within 1 month of presentation compared with SD-only (8.5%), MI-only (6.7%), and no MI or SD (1.7%). The mean number of days spent in hospital were similar across the four groups.

There were 14,351 patients with at least 2 years of follow-up data. Age distributions were similar, but a slightly higher proportion of females than males remained in the sample. Between 51.6% and 74.5% had a diagnosis at 2-years follow-up (vs. 11.7%–29.9% at baseline). The proportion of patients prescribed multiple medications and hospitalized remained highest in MI and SD. Patients without MI or SD had the fewest hospitalizations, but spent, on average, approximately 6–14 days longer in hospital than the patients from the other groups. Few individuals in any study group received HoNOS assessments within 2 years of first presentation (Table 3).

**Table 3.** Descriptive features of the sample with mood instability-only, sleep disturbance-only, mood instability and sleep disturbance, and no mood instability or sleep disturbance within 2-years of presenting to OHFT (*n* = 14,351).

	Mood instability-only		Sleep disturbance-only		Mood instability and sleep disturbance		No mood instability or sleep disturbance	
	(*n* = 627)		(*n* = 2,793)		(*n* = 835)		(*n* = 10,096)	
	*n*	%	*n*	%	*n*	%	*N*	%
Age								
16–25	81	12.92	248	8.88	90	10.78	1,000	9.90
26–35	190	30.30	816	29.22	286	34.25	2,962	29.34
36–45	190	30.30	848	30.36	242	28.98	2,916	28.88
46–55	140	22.33	712	25.49	180	21.56	2,252	22.31
56–65	26	4.15	169	6.05	37	4.43	966	9.57
Gender, female	373	59.49	1,512	54.14	491	58.8	5,480	54.28
Diagnosis								
Any	377	60.13	1,775	63.55	622	74.49	5,212	51.62
Organic	<5	–	15	0.54	5	0.60	131	1.30
Substance use	24	3.83	165	5.91	48	5.75	338	3.35
Schizophrenia and related	73	11.64	377	13.50	110	13.17	1,459	14.45
Mood	191	30.46	952	34.09	382	45.75	1,993	19.74
Anxiety	56	8.93	395	14.14	92	11.02	925	9.16
Behavioral syndrome	21	3.35	68	2.43	18	2.16	328	3.25
Personality	86	13.72	256	9.17	141	16.89	821	8.13
Mental retardation	<5	–	5	0.18	<5	–	18	0.18
Developmental	<5	–	12	0.43	<5	–	77	0.76
Behavioral/emotional	8	1.28	11	0.39	5	0.6	101	1.00
Unspecified	5	0.80	16	0.57	9	1.08	63	0.62
Medication								
1	<5	–	<5	–	<5	–	<5	–
2	188	29.98	777	27.82	184	22.04	2,431	24.08
3+	294	46.89	1,600	57.29	584	69.94	2,955	29.27
Any	483	77.03	2,377	85.11	768	91.98	5,389	53.38
Antidepressant	352	56.14	1,924	68.89	583	69.82	3,781	37.45
Antipsychotic	286	45.61	1,380	49.41	517	61.92	3,044	30.15
Mood-stabilizing antiepileptic	76	12.12	248	8.88	134	16.05	632	6.26
Sedative hypnotic	192	30.62	1,224	43.82	462	55.33	1,960	19.41
Lithium	49	7.81	172	6.16	99	11.86	393	3.89
Sedating antihistamine	9	1.44	104	3.72	38	4.55	122	1.21
Sympathomimetic	7	1.12	20	0.72	8	0.96	94	0.93
Amphetamine	<5	–	<5	–	<5	–	5	0.05
Events								
12–23	95	15.15	400	14.32	104	12.46	1,681	16.65
24–35	83	13.24	313	11.21	91	10.90	1,273	12.61
36+	320	51.04	1,579	56.53	553	66.23	4,550	45.02
Hospitalization								
Days in hospital, mean (SD)	88	34.38 (42.51)	510	38.25 (50.54)	298	36.42 (45.75)	903	48.77 (55.88)
1	63	10.05	364	13.03	205	24.55	629	6.23
2	22	3.51	83	2.97	55	6.59	175	1.73
3+	<5	–	63	2.26	38	4.55	99	0.98
HoNOS, mean (SD)	75	13.24 (11.60)	426	13.01 (10.60)	142	11.43 (6.73)	716	12.56 (6.99)

HoNOS=Health of the Nation Outcome Scale adjusted total score.

– Estimates unavailable due to small numbers.

### Prevalence of mood instability and sleep disturbance

The prevalence of MI at baseline was approximately 9.6% (95% CI: 9.3–9.9), was higher in females, and decreased with age. Approximately 26.3% (95% CI: 25.8–26.8) of patients presented with SD (sleep problems or insomnia). Prevalence of SD was higher in males and increased with age, except among the oldest age group for whom prevalence was lowest. The prevalence of sleep problems and insomnia were 24.9% (95% CI: 24.4–25.4) and 4.0% (95% CI: 3.8–4.3), respectively, and showed similar trends in gender and age-specific prevalence (Table 4).

**Table 4.** Total, gender-specific, and age-specific prevalence estimates for mood instability, sleep problems, insomnia, and combined sleep problems and insomnia.

	*n*	Mood instability			Sleep disturbance			Sleep problems			Insomnia		
		*n*	%	95% CI	*n*	%	95% CI	*n*	%	95% CI	*n*	%	95% CI
Total	31,391	3,006	9.58	9.25–9.90	8,243	26.26	25.77–26.75	7,809	24.88	24.40–25.35	1,267	4.04	3.82–4.25
Sex													
Female	17,204	1,700	9.88	9.44–10.33	4,347	25.27	24.62–25.92	4,139	24.06	23.42–24.70	639	3.71	3.43–4.00
Male	14,134	1,303	9.22	8.74–9.70	3,884	27.48	26.74–28.22	3,658	25.88	25.16–26.60	627	4.44	4.10–4.78
Age													
16–25	3,029	340	11.22	10.10–12.35	743	24.53	23.00–26.06	701	23.14	21.64–24.64	114	3.76	3.09–4.44
26–35	9,599	1,004	10.46	9.85–11.07	2,544	26.5	25.62–27.39	2,408	25.09	24.22–25.95	385	4.01	3.62–4.40
36–45	8,676	833	9.60	8.98–10.22	2,380	27.43	26.49–28.37	2,244	25.86	24.94–26.79	392	4.52	4.08–4.96
46–55	7,085	675	9.53	8.84–10.21	2,063	29.12	28.06–30.18	1,965	27.73	26.69–28.78	293	4.14	3.67–4.60
56–65	3,002	154	5.13	4.34–5.92	513	17.09	15.74–18.44	491	16.36	15.03–17.68	83	2.76	2.18–3.35

### Incidence rates of selected clinical features

After adjusting for multiple comparisons, the IRR of any psychiatric diagnosis was significantly increased in persons with MI and SD (IRR = 1.8, 05% CI: 1.7–2.0, *p* < 0.0001) and SD-only (IRR = 1.4, 95% CI: 1.4–1.5, *p* < 0.0001), but not MI-only (IRR = 1.2, 95% CI: 1.0–1.3, *p* < 0.007) compared to those without MI or SD (Table 5). The IRR for substance use was strongest among those with SD-only (IRR = 1.5, 95% CI: 1.3–1.8, *p* < 0.001), whereas the IRR for mood disorder was strongest for those with MI and SD (IRR = 1.8, 95% CI: 1.6–2.0, *p* < 0.0001). SD-only was associated with significantly increased IRR of antidepressants and sedative-hypnotics. MI and SD showed significantly increased IRR for lithium, and had the highest IRR of first hospitalization within 2-years follow-up (IRR = 5.32, 95% CI: 4.7–6.1, *p* < 0.0001).

**Table 5.** Estimated incidence rate ratios of diagnoses, prescribed medications, and first hospitalization within 2 years of presenting to OHFT in mood instability-only, sleep disturbance-only, and mood instability and sleep disturbance versus no mood instability or sleep disturbance.

	Mood instability-only			Sleep disturbance-only			Mood instability and sleep disturbance		
	IRR	95% CI	*p*-value	IRR	95% CI	*p*-value	IRR	95% CI	*p*-value
Diagnosis									
Any	1.16	1.04–1.28	0.007	1.44	1.36–1.52	<0.0001*	1.81	1.66–1.96	<0.0001*
Organic	1.16	0.43–3.13	0.772	0.93	0.55–1.56	0.772	1.45	0.59–3.53	0.414
Substance use	1.45	0.97–2.17	0.071	1.50	1.26–1.79	<0.0001*	1.57	1.20–2.06	0.001*
Schizophrenia and related	1.18	0.93–1.49	0.177	1.35	1.20–1.51	<0.0001*	1.57	1.29–1.90	<0.0001*
Mood	1.04	0.90–1.20	0.617	1.41	1.31–1.53	<0.0001*	1.77	1.59–1.97	<0.0001*
Anxiety	1.26	0.96–1.64	0.089	1.31	1.17–1.47	<0.0001*	1.50	1.22–1.86	<0.0001*
Behavioral syndrome	1.11	0.89–1.38	0.362	1.16	1.00–1.33	0.042	1.57	1.31–1.88	<0.0001*
Personality	0.78	0.50–1.19	0.247	1.11	0.86–1.43	0.442	1.38	0.86–2.21	0.186
Mental retardation	–	–	–	1.2	0.45–3.24	0.713	0.36	0.05–2.71	0.301
Developmental	0.79	0.32–1.95	0.610	0.88	0.49–1.58	0.672	0.63	0.23–1.73	0.367
Behavioral and emotional	1.79	0.87–3.67	0.109	0.77	0.42–1.39	0.380	2.23	0.91–5.48	0.072
Unspecified	2.14	0.86–5.33	0.093	0.98	0.57–1.70	0.941	0.77	0.38–1.55	0.465
Medication									
Any	2.26	2.06–2.48	<0.0001*	3.58	3.41–3.76	<0.0001*	7.04	6.53–7.59	<0.0001*
Antidepressant	1.73	1.55–1.93	<0.0001*	2.34	2.21–2.47	<0.0001*	3.21	2.94–3.50	<0.0001*
Antipsychotic	1.56	1.39–1.77	<0.0001*	1.94	1.82–2.07	<0.0001*	3.13	2.85–3.44	<0.0001*
Mood-stabilizing antiepileptic	1.67	1.32–2.12	<0.0001*	1.44	1.25–1.67	<0.0001*	1.31	1.09–1.58	0.004
Sedative hypnotic	1.31	1.13–1.52	<0.0001*	2.35	2.19–2.52	<0.0001*	4.73	4.27–5.23	<0.0001*
Lithium	1.03	0.76–1.38	0.867	1.09	0.91–1.31	0.325	1.78	1.43–2.22	<0.0001*
Sedating antihistamine	1.14	0.58–2.24	0.708	1.21	0.93–1.57	0.150	1.68	1.17–2.42	0.005
Sympathomimetic	1.29	0.60–2.78	0.514	1.84	1.14–2.99	0.012	1.48	0.72–3.04	0.288
Amphetamine	–	–	–	–	–	–	–	–	–
First hospitalization	1.61	1.30–2.01	<0.0001*	1.98	1.77–2.21	<0.0001*	5.32	4.67–6.07	<0.0001*

* Indicates significance in relation to the Bonferroni-Holm critical value.

– represents estimates unavailable due to small numbers.

## Discussion

### Summary of findings

This study examined clinical outcomes among patients presenting with MI and/or SD to secondary mental health services, and provides further evidence that MI and SD are commonly found in a wide range of psychiatric disorders [17–19].

We observed a slightly lower prevalence of MI (9.58%) than a previous study using the MI application in data from SLaM NHS Trust (12.1%) [8]. This is likely because Patel et al. restricted their sample to affective, psychotic or personality disorders, many of which are often characterized by MI [20–22], whereas our sample included all patients, irrespective of diagnosis. We found a higher prevalence of MI in females and young people, consistent with Patel et al. [8] and with patterns observed in the general population [6].

The observed patterns in age and gender-specific prevalence of SD align with the wider literature, where increased prevalence is associated with age and female gender [23,24]. However, the overall prevalence of SD observed in this study was considerably lower than expected for a clinical psychiatric populations [25], which is likely due to the limited range of terms identified by the SD NLP applications.

Regardless of the presence of MI and/or SD, the most frequent diagnoses were mood, anxiety, personality and schizophrenia and related disorders. Although there were some differences between the study groups, there were no clear trends in their relationships with specific diagnostic categories. This appears to support the hypothesis that MI and SD are transdiagnostic processes given their presence in nearly all classes of primary mental disorders.

The proportion of patients with a recorded diagnosis was low despite continued care; only approximately 56% of patients had a diagnosis within 2 years. The diagnosis field is not compulsory and located within a separate part of the EHR, and therefore may not be routinely filled in by health care providers. Many assessments are not conducted by doctors, and non-medical health care providers may feel less comfortable or able to give a diagnosis. An examination of records from SLaM found that only 82%–93% of patients were ever assigned a primary diagnosis [9]. Since we restricted our study to only primary psychiatric conditions within the past 2-years, a subset of patients with other diagnoses, such as neurological conditions, were excluded.

Notably, the prevalence of sleep disorder diagnoses in this study was negligible (<0.5%), and considerably lower than reported in the wider literature [25]. The low frequency may reflect a common view that SD is an epiphenomenon of the primary psychiatric disorder [26]. If sleep problems are viewed as secondary symptoms, then sleep disorders may be underdiagnosed in primary psychiatric patients, even when SD is identified. Nevertheless, the proportion of sedative-hypnotic prescriptions was nearly two times higher in SD-only than MI-only, which may suggest recognition of SD as an important focus of treatment. Given extensive evidence that SD may be a maintaining factor in mental illness, a shift toward better recognition of SD will provide important benefits to treatment.

Individuals with MI and SD showed very high service use. Over 85% of people with MI and SD had three or more contacts with services within their first month of presentation, and over 65% had 36 or more visits over the 2-year period, equating to approximately more 1.5 visits per month. Interestingly, while patients without MI or SD had the fewest hospitalizations, they spent, on average, 1–2 weeks longer in hospital than patients from the other three groups. There may be several possible explanations for this finding. One possible reason may be the higher proportion of personality disorders in the patients with MI and/or SD compared with those without either feature. There is concern that hospitalization may be counterproductive in personality disorders [27], which may account for slightly shorter hospital duration in these groups compared to people without MI or SD. Further MI and SD are modifiable, and their stabilization may lead to improvements that allow for shorter hospitalizations [28].

An unexpectedly small proportion of individuals in the sample had HoNOS ratings, and the low completion rate limits the conclusions that can be drawn from the HoNOS-related findings. HoNOS is a main routine outcome measure of health and social functioning in patients with mental illness in England, as set by the NHS [15], and comprise part of the Mental Health Minimum Data Set (MHSDS). The absence of these data may have important implications for resource allocation to OHFT and also for the implementation of mental health care in England.

This study adds to evidence that psychiatric symptoms like MI can be identified from EHRs using NLP applications with reasonable accuracy. Previous validation of the MI applications in a sample of EHRs from patients who presented to SLaM, showed a sensitivity of approximately 91%, and positive predictive values ranging from approximately 46%–73% for mood, affective, and emotional instability [8]. In our study, the combined accuracy of these three applications showed a similar specificity (92%), but considerably higher positive predictive value (94%). The other applications for identifying sleep problems, insomnia, and prescribed medications also showed good accuracy, with sensitivity estimates ranged between 80% and 86%, and positive predictive values between 82% and 92%.

### Limitations

Some limitations to this study are acknowledged. First, EHRs represent only information documented during service provision and missing data cannot be quantified. For example, it is unclear whether a missing diagnostic code reflects the absence of a diagnosis or just no record of a diagnosis within that field.

MI, SD, and prescribed medications were identified from clinical progress notes and correspondence, and therefore, reflect only information documented during routine care. Specific symptoms may not be discussed uniformly or routinely in each patient, but rather in relation to a number of other factors, such as the patient’s diagnosis or medication. For example, MI may be overlooked within disorders for which these symptoms are seen as uncommon.

Additionally, NLP applications have some degree of inaccuracy, which may have introduced bias into the prevalence and IRR estimates. Although some validation of the applications was carried out in this study, it was based on a relatively small number of manual annotations by a single human annotator due to time and resource constraints. Validation could be improved by assessing a larger sample of documents, and using more than one annotator to permit estimation of inter-rater reliability.

Further, the outputs of the NLP applications are dependent on the terms that they are intended to identify. For example, the low prevalence of SD observed in this study may reflect somewhat limited terms identified by the NLP applications for SD. The applications showed reasonably accuracy in the identification of mentions similar to “sleep problems” and “insomnia,” but they did not identify other words or phrases that could also indicate issues with sleep, such as “somnolence” or unusual behaviors during sleep.

Finally, severity of MI and SD could not be assessed from the NLP applications used in this study. Although, ideally, MI and SD are routinely assessed in care, this may not be the case, and there is little standardization between clinical assessments and limited deployment of standardized measures routinely used to assess these features.

### Future directions

The results of the current study highlight important avenues for future work. Foremost, interventional research may help to better understand and improve the effectiveness of transdiagnostic treatments (both pharmacological and psychosocial treatments) of MI and SD. For example, cognitive behavioral therapy (CBT) for insomnia delivered at hospital admission has been shown to considerably reduce insomnia and marginally improve psychological well-being after 2 weeks [28]. Further work could aim to produce adaptations to CBT with the aim of improving MI in addition to sleep, and for delivery outside of acute care.

Many pharmacological treatments that target mood disturbances also have secondary effects that may exacerbate or improve symptoms of SD. For example, insomnia is among one of the most frequently reported adverse events of fluoxetine, while trazodone has been found to improve sleep symptoms in patients with depression plus insomnia [29]. Further research is needed to clarify which treatments may be most effective in persons with both MI and SD.

Future research should also seek to clarify factors that influence clinical outcomes, such as hospitalization, in individuals with MI and SD. Early interventions targeted at modifiable risk-factors may help to reduce complications and improve quality of life in persons with mental illness.

Finally, EHRs hold a wealth of clinically relevant information and NLP applications offer a novel method to examine these data. More work is needed to refine and validate NLP methods for eliciting symptoms from EHRs. Future research may also seek to establish whether the accuracy of the NLP applications could be improved by altering thresholds for the presence or absence of each feature. This study established the presence of MI based on one or more mentions of MI within documents within the first month of presentation. Future work may seek to determine if accuracy can be improved when, for example, mentions of the feature in two or more documents are required.

Given growing evidence that MI and SD are transdiagnostic features of psychiatric disorders, and their important impact on clinical outcomes and functioning, it is important they are assessed routinely in clinical care. Regular ongoing monitoring of mood and sleep are recommended for patients with mood and anxiety disorders [30]. Brief standardized assessments of these symptoms, such as those included on the True Colors remote monitoring platform (https://oxfordhealth.truecolours.nhs.uk) may be useful tools for health care providers. Evidence-based clinical practice guidelines for the assessment and treatment of sleep disorders are available [31] to improve the standardization of assessment and care for patients.

## Conclusions

The findings of this study suggest that MI and SD may contribute to the high cost of mental disorders, especially when they co-occur. MI and SD are present in a range of psychiatric disorders and associated with increased use of services and prescribed medications, and significantly increased risk of hospitalization. These findings have important implications for clinical practice. One possible reason for the poor outcomes associated with MI and SD may be that they are often overlooked during care. Both MI and SD are often seen as epiphenomena of the underlying disorder. However, growing evidence that they are transdiagnostic features and involved in the origins and maintenance of mental disorders implies that routine assessment and prompt and ongoing treatment may help to improve prognosis in individuals with a wide range of mental disorders.

## Data Availability

The data that support the findings of this study are available from NIHR Biomedical Research Center. Restrictions apply to the availability of these data, which were used under license for this study. Data are available from the authors with the permission of NIHR Biomedical Research Center.

## Supplementary material

For supplementary material accompanying this paper visit http://dx.doi.org/10.1192/j.eurpsy.2020.39. click here to view supplementary material
