# Supplementary material for: Prevalence and incidence of clinical outcomes in patients presenting to secondary mental health care with mood instability and sleep disturbance
Source: Eur Psychiatry. 2020 Apr 27;63(1):e59. doi: 10.1192/j.eurpsy.2020.39 (PMC7355164; doi:10.1192/j.eurpsy.2020.39)
Supplement: Supplementary file 1 [file S0924933820000395sup001.docx]

**Supplementary Materials**

Supplementary Table 1. Classification of psychotropic medications identified by the natural language processing application.

| Antidepressants | Antipsychotics | Mood stabilising antiepileptics | Sedative-hypnotics | Amfetamines | Sympathomimmetics | Antihistamines |
| --- | --- | --- | --- | --- | --- | --- |
| Agomelatine | Amisulpride | Carbamazepine | Alprazolam | Dexamfetamine | Atomoxetine | Promethazine |
| Amoxapine | Amitriptyline | Lamotrigine | Bromazepam | Lisdexamfetamine | Methylphenidate |  |
| Bupropion | Aripiprazole | Sodium valproate | Buspirone |  | Modafinil |  |
| Citalopram | Asenapine | Valproic acid | Chloral hydrate |  |  |  |
| Clomipramine | Chlorpromazine |  | Chlordiazepoxide |  |  |  |
| Dosulepin | Clozapine |  | Clobazam |  |  |  |
| Doxepin | Droperidol |  | Clomethiazole |  |  |  |
| Duloxetine | Flupentixol |  | Clonazepam |  |  |  |
| Escitalopram | Fluphenazine |  | Diazepam |  |  |  |
| Fluoxetine | Haloperidol |  | Flunitrazepam |  |  |  |
| Fluvoxamine | Imipramine |  | Flurazepam |  |  |  |
| Isocarboxazid | Lurasidone |  | Loprazolam |  |  |  |
| Lofepramine | Olanzapine |  | Lorazepam |  |  |  |
| Mianserin | Paliperidone |  | Lormetazepam |  |  |  |
| Mirtazapine | Periciazine |  | Melatonin |  |  |  |
| Moclobemide | Perphenazine |  | Midazolam |  |  |  |
| Nefazodone | Pimozide |  | Nitrazepam |  |  |  |
| Nortriptyline | Pipotiazine |  | Oxazepam |  |  |  |
| Reboxetine | Prochlorperazine |  | Zolpidem |  |  |  |
| Sertraline | Promazine |  | Zopiclone |  |  |  |
| Tranylcypromine | Questiapine |  |  |  |  |  |
| Trazodone | Risperidone |  |  |  |  |  |
| Trimipramine | Sertindole |  |  |  |  |  |
| Venlafaxine | Sulpiride |  |  |  |  |  |
|  | Thioridazine |  |  |  |  |  |
|  | Ziprasidone |  |  |  |  |  |
|  | Zuclopenthixol |  |  |  |  |  |
